# A systematic review and meta-analysis on the risk of migraine in patients with allergic rhinitis

**DOI:** 10.3389/fmed.2026.1766176

**Published:** 2026-04-02

**Authors:** Jiaqi Wu, Chao Wang, Fanqiang Meng, Hebo Wang

**Affiliations:** 1 Hebei North University Graduate School, Zhangjiakou, China; 2 Department of Neurology, Hebei General Hospital, Shijiazhuang, China; 3 Graduate School of North China University of Science and Technology, Tangshan, China; 4 Hebei Provincial Key Laboratory of Cerebral Networks and Cognitive Disorders, Shijiazhuang, China

**Keywords:** allergic rhinitis, meta-analysis, migraine, risk factor, systematic review

## Abstract

**Background:**

Allergic rhinitis (AR) and migraine are common chronic conditions that significantly impact patients’ quality of life. Although observational studies suggest a potential link between AR and increased migraine risk, evidence remains inconsistent.

**Objective:**

To systematically evaluate the association between AR and migraine risk through a comprehensive meta-analysis.

**Methods:**

Databases including PubMed, Web of Science, Scopus, and CNKI were systematically searched up to July 2025. Observational studies investigating the association between AR and migraine were included. Data extraction, quality assessment, and meta-analysis were performed following PRISMA guidelines. Random-effects models were utilized, and subgroup analyses were conducted (by study design, age group, and effect measure). Sensitivity analyses (exclusion of cross-sectional studies, restriction to large-sample studies, use of unadjusted estimates, and leave-one-out procedures) were conducted to assess robustness. Publication bias was evaluated using funnel plots, trim-and-fill analysis, and Egger’s regression test. Additional analyses included meta-regression to explore sources of heterogeneity, cumulative meta-analysis to assess temporal trends, and conversion of relative effects to absolute risk estimates.

**Results:**

Ten studies involving more than 4.8 million participants met the inclusion criteria. Pooled results indicated a significantly elevated risk of migraine among individuals with AR (OR = 2.75; 95% CI, 1.80–4.19; I^2^ = 99%). Subgroup analyses demonstrated consistent findings across study designs, age groups, and analytical approaches. Sensitivity analyses indicated stable results, with no single study markedly influencing the pooled estimate. Meta-regression suggested that study design, age group, and sample size explained part of the heterogeneity. Cumulative analysis showed that early small-sample studies overestimated the association, while larger recent studies stabilized the effect at 2–3 fold risk. Absolute effect estimates indicated an excess of 23–82 migraine cases per 1,000 individuals with AR, with population attributable fractions ranging from 6.8 to 18.1%.

**Conclusion:**

Allergic rhinitis is significantly associated with an increased risk of migraine. Clinicians should be aware of this association and consider integrated management strategies for patients with comorbid AR and migraine.

**Systematic review registration:**

INPLASY2025110052.

## Introduction

Allergic rhinitis (AR) is one of the most prevalent chronic allergic diseases, affecting up to 40% of the global population, characterized by symptoms such as nasal congestion, rhinorrhea, sneezing, and nasal pruritus, significantly impairing daily life and productivity (1, 2). Migraine, a common primary headache disorder, impacts approximately 15% of the adult population worldwide and is recognized as one of the leading causes of disability, with considerable socioeconomic burden (3, 4). Emerging epidemiological evidence indicates that AR and migraine frequently coexist, suggesting potential shared pathogenic mechanisms and inflammatory pathways (5–7).

Previous observational studies have reported that individuals with allergic disorders, especially allergic rhinitis, may have a higher prevalence and risk of developing migraine compared to those without allergic conditions (8–11). Clinical and experimental studies have proposed several mechanisms underlying the link between AR and migraine, highlighting the role of shared inflammatory mediators and neural pathways. Neurogenic inflammation mediated by calcitonin gene-related peptide (CGRP) and vasoactive intestinal peptide (VIP) is a critical mechanism implicated in migraine pathophysiology (3, 12, 13). Interestingly, elevated levels of CGRP have been identified in nasal secretions and plasma samples from AR patients, suggesting a potential overlap in inflammatory and neural pathways between these two disorders (14, 15).

Despite the biological plausibility, the results of existing observational studies have been inconsistent. While some large-scale cohort studies identified a clear association between AR and migraine (16, 17), others reported modest or no significant relationships, possibly due to variations in study populations, diagnostic criteria, or methodological differences (18, 19). Furthermore, genetic studies have provided conflicting evidence on whether shared genetic factors underpin the association between AR and migraine, with recent Mendelian randomization analyses suggesting non-genetic mechanisms as primary contributors (20, 21).

Given these uncertainties and the considerable clinical implications for patient management, a comprehensive assessment of the current evidence is required. Therefore, the objective of this systematic review and meta-analysis was to clarify the association between allergic rhinitis and migraine, synthesizing the latest available observational data and providing robust evidence to guide clinical practice.

## Methods

### Protocol and registration

This systematic review and meta-analysis was conducted following the Preferred Reporting Items for Systematic Reviews and Meta-Analyses (PRISMA) 2020 guidelines (22). The initial literature search was performed in July 2025 to define the scope of the review. The full study protocol was subsequently registered with INPLASY (Registration Number: INPLASY2025110052) in November 2025, prior to formal data extraction and analysis. While this represents a retrospective registration, all analytical plans were pre-specified in the registered protocol.

### Search strategy and information sources

A comprehensive literature search was conducted systematically across four electronic databases: PubMed, Web of Science Core Collection, Scopus, and China National Knowledge Infrastructure (CNKI). The final search was performed on July 7, 2025, without restrictions on language, geographic location, or publication status. The detailed search strategies employed for each database were as follows:

*PubMed*: (‘allergic rhinitis’[Major Topic] OR ‘allergic rhinitis’[Title/Abstract]) AND (‘migraine*’[Title/Abstract] OR ‘Migraine Disorders’[Major Topic]).
*Web of Science*: ‘TS = (“allergic rhinitis” AND migraine)’, yielding 57 records.
*Scopus*: ‘TITLE-ABS-KEY(“allergic rhinitis” AND migraine)’, yielding 247 records.
*CNKI*: SU = (‘allergic rhinitis’) AND SU = (‘migraine’), yielding 53 records.

Additionally, reference lists of eligible studies and relevant systematic reviews were manually reviewed to identify potential additional studies.

### Eligibility criteria

Studies were considered eligible if they met the following criteria:

*Population*: Patients diagnosed with allergic rhinitis (AR), confirmed by clinical criteria, physician diagnosis, skin prick test (SPT), Immunoglobulin E (IgE) positivity, or validated diagnostic codes (ICD-9/10).
*Comparator*: Individuals without allergic rhinitis.
*Outcome*: The primary outcome was the occurrence or diagnosis of migraine, defined by International Headache Society (IHS) criteria or standard diagnostic codes (ICD-9/10). Studies reporting migraine prevalence, incidence, or adjusted risk estimates (Odds Ratio \(OR), Hazard Ratio \(HR), or Relative Risk \(RR)) were included.
*Study design*: Observational studies, including cohort, cross-sectional, and case–control studies. Studies without clear diagnostic criteria, case reports, animal studies, reviews, letters, or conference abstracts without sufficient data were excluded.

### Study selection

Initial screening based on titles and abstracts was performed independently by two reviewers using EndNote software (X20). Full-text assessments of potentially eligible articles were conducted independently, with discrepancies resolved by consensus or a third reviewer.

### Data extraction

Two independent reviewers WJQ and WC extracted data using a standardized form, recording the following information: author, publication year, country, study design, sample size, diagnostic criteria for AR and migraine, the number of migraine cases in AR and non-AR groups, adjusted effect sizes (aOR, aHR) and corresponding 95% confidence intervals (CI), and covariates used in adjustment. Discrepancies were resolved through discussion or adjudication by a third reviewer.

### Quality assessment

Quality assessment was conducted independently by two reviewers using the Newcastle-Ottawa Scale (NOS) for cohort and case–control studies, and the Joanna Briggs Institute (JBI) Critical Appraisal Checklist for cross-sectional studies (23, 24). NOS ratings were categorized as low (0–3), moderate (4–6), or high (7–9). For cross-sectional studies, methodological quality was appraised using the JBI Critical Appraisal Checklist, which includes eight items evaluating sample frame, sample size, subject and setting description, validity of exposure and outcome measurement, identification and management of confounders, and appropriateness of statistical analysis. Each item was rated as Yes/No/Unclear/Not applicable, and an overall quality level (High/Moderate) was assigned accordingly. Cohort and case–control studies were judged as low (0–3), moderate (4–6), or high (7–9) quality according to NOS scores, while cross-sectional studies were judged as high quality if ≥70% of items were “Yes,” moderate if 50–69% were “Yes,” and low if <50% were “Yes.”

### Data synthesis and statistical analysis

Statistical analyses were performed using Review Manager (RevMan, version 5.4) and R software (meta and metafor packages). Adjusted odds ratios (aORs) and hazard ratios (aHRs) were considered directly if available; otherwise, crude ORs were calculated from 2 × 2 contingency tables. HRs reflect time-to-event associations while ORs reflect odds associations. Due to anticipated heterogeneity, the DerSimonian-Laird random-effects model was applied to pool effect estimates.

Heterogeneity was assessed using Cochran’s Q statistic and quantified by the I^2^ statistic, where I^2^ values of 25, 50, and 75% corresponded to low, moderate, and high heterogeneity, respectively. Subgroup analyses were planned based on age groups, study designs (cohort, case–control, cross-sectional) and effec measure. Sensitivity analyses were conducted by removing individual studies sequentially to test result stability.

Publication bias was visually examined through funnel plots and statistically assessed by Egger’s regression test, with significance set at *p* < 0.05.

To test the robustness of the findings, several sensitivity analyses were conducted, including exclusion of cross-sectional studies, restriction to large-sample studies, and comparison of adjusted versus unadjusted effect estimates. Leave-one-out analyses were further performed by sequentially excluding each study to assess its individual influence on the pooled effect.

To explore potential sources of heterogeneity, we performed multivariate meta-regression and τ^2^ decomposition analyses, examining study-level covariates such as study design, age group, sample size, and geographic region.

A cumulative meta-analysis was also conducted by ordering studies chronologically by publication year to evaluate temporal trends and the stability of pooled estimates as evidence accumulated.

Absolute effect measures were derived by applying pooled relative effect estimates to baseline prevalence scenarios of migraine in different populations. From these conversions, risk differences, population attributable fractions (PAFs), and number needed to treat–like (NNT-like) values were calculated to enhance clinical and public health interpretability.

### Ethical considerations

As this study is a systematic review and meta-analysis utilizing only published aggregate data, it does not involve direct interaction with human participants. Therefore, ethical approval and informed consent were not required.

## Results

### Study selection

A total of 3,754 records were identified through comprehensive searches across four electronic databases, including PubMed (*n* = 3,397), Web of Science (*n* = 57), Scopus (*n* = 247), and CNKI (*n* = 53). After removal of duplicate records (*n* = 291) and records excluded for other reasons (*n* = 1,829; including animal studies, conference abstracts, reviews, irrelevant outcomes, or non-English/Chinese publications), 1,634 unique articles remained for screening. 1,634 unique articles underwent title and abstract screening, during which 1,571 records were excluded because of irrelevance to the research topic, inappropriate study design (e.g., reviews, animal studies), or failure to meet inclusion criteria.

Subsequently, 63 potentially eligible full-text articles were retrieved and thoroughly evaluated. Among these, 53 studies were further excluded due to the following reasons: irrelevant exposure or outcome measures (*n* = 18), review articles or case reports (*n* = 15), insufficient quantitative data for meta-analysis (*n* = 12), duplicated cohorts or overlapping data from the same population (*n* = 5), and studies conducted in animal models (*n* = 3).

**Figure 1:**
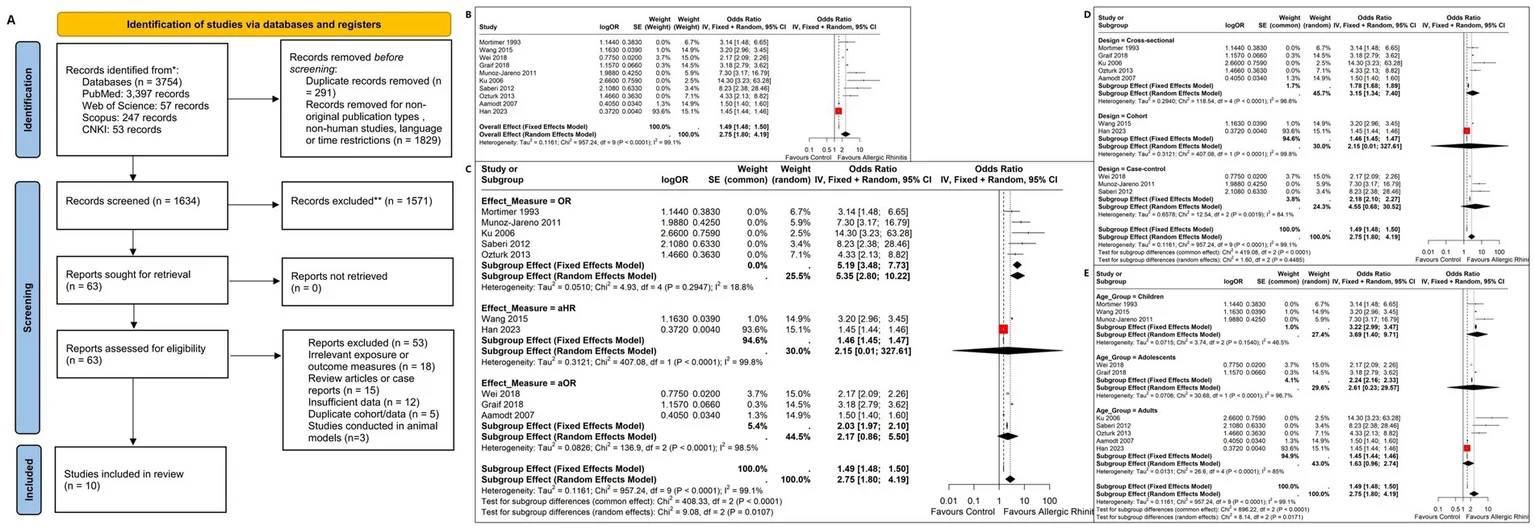
**(A)** PRISMA 2020 flow diagram; **(B)** overall pooled association (forest plot); **(C)** subgroup analysis by effect measure (forest plot); **(D)** subgroup analysis by study design (forest plot); **(E)** subgroup analysis by age group (forest plot).

Ultimately, a total of 10 studies met all predefined eligibility criteria and were included in both qualitative synthesis and quantitative meta-analysis. The detailed stepwise selection process is presented clearly in the PRISMA 2020 flow diagram (Figure 1A).

### Characteristics of included studies

Ten studies met the inclusion criteria, comprising data from a total of 4,773,230 participants across various geographic regions, including Europe, America, Asia, and the Middle East. These studies consisted of five cross-sectional studies, three case–control studies, and two cohort studies. The study characteristics are summarized in detail in Table 1 (16–18, 25–31).

**Table 1:** Characteristics of studies included in the meta-analysis.

First author (year)	Country/Region	Study design	Population and age	AR diagnostic criteria	Migraine diagnostic criteria	AR^+^ sample size/events	AR^−^ sample size/events	Reported effect size (95% CI)
Mortimer (1993) (25)	UK	Cross-sectional (Community)	School children (3–11 y)	Parental questionnaire + clinical diagnosis	IHS 1988	82/10	1,015/43	OR = 3.14 (1.48–6.64)
Ku (2006) (26)	USA	Cross-sectional (Clinic)	Adults (≥18 y)	Skin prick test / RAST	IHS 1988	76/26	57/2	OR = 14.30 (3.23–63.34)
Aamodt (2007) (17)	Norway	Cross-sectional (HUNT2)	General population (≥20 y)	Self-reported “hay fever”	IHS revised algorithm	8,969/1,560	38,061/4,215	aOR = 1.50 (1.40–1.60)
Saberi (2012) (27)	Iran	Case–control	ENT patients (≥18 y)	Skin prick test ≥ 3 mm (8 allergens)	IHS 1988	46/17	60/3	OR = 8.23 (2.38–28.42)
Muñoz-Jareño (2011) (28)	Spain	Case–control	Outpatients (5–15 y)	ISAAC questionnaire (RC)	ICHD-II	90/61	126/29	OR = 7.30 (3.20–16.60)
Wang (2015) (29)	Taiwan	Retrospective cohort (NHIRD)	National child population (≤18 y)	ICD-9-CM ≥ 1 diagnostic record	First clinical visit/hospitalization	461,850/2,823	460,718/860	aHR = 3.20 (2.97–3.46)
Ozturk (2013) (18)	Pakistan	Cross-sectional (ENT clinic)	Clinical patients (12–65 y)	Skin prick test + IgE	IHS 2004	80/40	80/15	OR = 4.33 (2.13–8.83)
Wei (2018) (30)	Taiwan	Case–control (NHIRD)	Children/adolescents (7–18 y)	ICD-9 prior records	Clinical visits/hospitalization	16,130/6,999	64,520/17,920	aOR = 2.17 (2.09–2.26)
Graif (2018) (16)	Israel	Cross-sectional (Military recruitment)	Youth (17 y)	Medical records + nasal medication history	Neurologist-confirmed	5,239/331	108,432/1,789	aOR = 3.18 (2.80–3.63)
Han (2023) (31)	Korea	Retrospective cohort (NHIS)	Adults undergoing routine check-ups (≥20 y)	ICD-10 ≥ 3 diagnostic records	First diagnosis	463,510/95,607	3,144,089/412,756	aHR = 1.45 (1.44–1.46)

AR, allergic rhinitis; OR, odds ratio; CI, confidence interval; NOS, Newcastle-Ottawa Scale; JBI, Joanna Briggs Institute.

### Quality assessment of included studies

Quality appraisal indicated predominantly high-to-moderate study quality. Both cohort studies scored 9/9 (high). Among the three case–control studies, two scored 6/9 (moderate) and one 7/9 (high). The JBI checklist to assess the quality of five cross-sectional studies included in the meta-analysis, with Ku (26), Aamodt (17) and Graif (16) rated as high quality due to meeting all eight assessment criteria while Mortimer (25) and Ozturk (18) were classified as moderate quality owing to the failure to identify confounding factors and adopt corresponding adjustment strategies, alongside an inadequate sample size for both studies. Detailed ratings, including cross-sectional study appraisals, are presented in Tables 2, 3.

**Table 2:** Quality assessment of cohort and case–control studies (NOS).

Study	Study Design	Selection (0–4 ★)	Comparability (0–2 ★)	Outcome/Exposure Assessment (0–3 ★)	Total Score	Quality Level
Wang, 2015 (29)	Cohort	★★★★	★★	★★★	9/9	High
Han, 2023 (31)	Cohort	★★★★	★★	★★★	9/9	High
Saberi, 2012 (27)	Case–control	★★★	★	★★	6/9	Moderate
Muñoz-Jareño, 2011 (28)	Case–control	★★★	★	★★	6/9	Moderate
Wei, 2018 (30)	Case–control	★★★	★★	★★	7/9	High

NOS total score: 0–3 low, 4–6 moderate, 7–9 high quality.

**Table 3:** Quality assessment of cross-sectional studies (JBI checklist).

Study (Year)	Q1. Sample frame appropriate	Q2. Sample size adequate	Q3. Subjects & setting described	Q4. Exposure measured validly	Q5. Outcome measured validly	Q6. Confounding factors identified	Q7. Strategies for confounding	Q8. Statistical analysis appropriate	Overall risk of bias (Quality Level)
Mortimer (1993) (25)	Yes 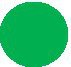	No 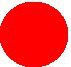	Yes 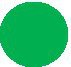	Yes 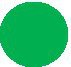	Yes 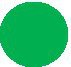	No 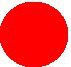	No 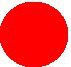	Yes 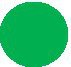	Moderate
Ku (2006) (26)	Yes 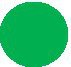	No 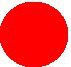	Yes 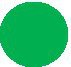	Yes 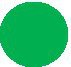	Yes 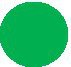	Yes 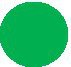	Yes 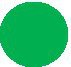	Yes 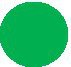	High
Aamodt (2007) (17)	Yes 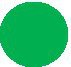	Yes 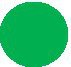	Yes 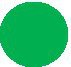	Yes 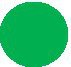	Yes 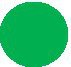	Yes 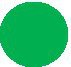	Yes 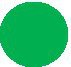	Yes 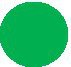	High
Ozturk (2013) (18)	Yes 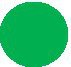	No 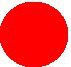	Yes 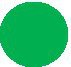	Yes 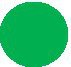	Yes 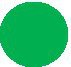	No 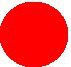	No 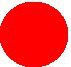	Yes 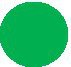	Moderate
Graif (2018) (16)	Yes 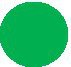	Yes 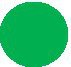	Yes 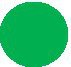	Yes 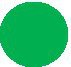	Yes 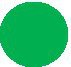	Yes 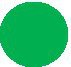	Yes 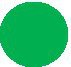	Yes 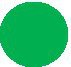	High

### Overall association between allergic rhinitis and migraine

The meta-analysis of the 10 included studies demonstrated a significant positive association between allergic rhinitis (AR) and the risk of migraine (Figure 1B). Using a random-effects model, the pooled odds ratio (OR) was 2.75 (95% CI, 1.80–4.19), indicating that patients with allergic rhinitis had approximately a 2.75-fold increased risk of developing migraine compared to individuals without allergic rhinitis. A high degree of heterogeneity was detected (I^2^ = 99%, *p* < 0.0001), supporting the use of the random-effects model. Given that Odds Ratios (ORs) may overestimate the risk when the outcome is not rare, we performed a sensitivity analysis by converting ORs to Relative Risks (RRs). As shown in Supplementary Figures S1A–C, the pooled RR was 2.46 (95% CI, 1.62–3.74), which is slightly lower than the pooled OR but still indicates a significant positive association. Additionally, separate analyses of cohort studies reporting Hazard Ratios (HRs) yielded consistent directions of effect. These results suggest that while the magnitude of the association varies by metric, the increased risk of migraine associated with allergic rhinitis is robust across different statistical measures.

### Subgroup analyses

#### Subgroup by effect measure

In subgroup analyses stratified by the type of effect measure (Figure 1C), studies reporting crude odds ratios (OR) showed the strongest association (OR = 5.35; 95% CI, 2.80–10.22), while studies reporting adjusted hazard ratios (aHR) showed a wide confidence interval spanning the null value (aHR = 2.15; 95% CI, 0.01–327.61). Similarly, studies using adjusted odds ratios (aOR) indicated a positive but statistically non-significant trend (aOR = 2.17; 95% CI, 0.86–5.50). Significant heterogeneity was present within all subgroups, particularly within studies reporting aHR (I^2^ = 99.8%) and aOR (I^2^ = 98.5%). The test for subgroup differences was statistically significant (χ^2^ = 9.08, df = 2, *p* = 0.011), and the effect measure explained approximately 42.7% of the overall heterogeneity.

#### Subgroup by study design

Subgroup analysis by study design (Figure 1D) showed consistent results across different designs. The pooled OR from cross-sectional studies was 3.15 (95% CI, 1.34–7.40), cohort studies showed a pooled estimate of 2.15 (95% CI, 0.01–327.61), and case–control studies demonstrated a pooled estimate of 4.55 (95% CI, 0.68–30.52). Despite differences in magnitude, all designs consistently indicated a significantly increased risk, with substantial heterogeneity within each subgroup (cross-sectional I^2^ = 96.6%; cohort I^2^ = 99.8%; case–control I^2^ = 84.1%). The test for subgroup differences was not statistically significant (χ^2^ = 1.60, df = 2, *p* = 0.45), suggesting that study design explained little of the observed heterogeneity, despite accounting for about 43.8% of the Q statistic.

#### Subgroup by age group

When analyzed by age groups (Figure 1E), children showed a significant positive association between allergic rhinitis and migraine risk (OR = 3.69; 95% CI, 1.40–9.71). Although adolescents also exhibited a positive association (OR = 2.61; 95% CI, 0.23–29.57), this result was not statistically significant. In the adult population, the pooled OR was 1.63 (95% CI, 0.96–2.74), which was also not statistically significant. Adult populations demonstrated a lower pooled OR of 1.63 (95% CI, 0.96–2.74). Substantial heterogeneity was noted across all age categories (children I^2^ = 46.5%; adolescents I^2^ = 96.7%; adults I^2^ = 85%). The test for subgroup differences was statistically significant (χ^2^ = 8.14, df = 2, *p* = 0.017), and age group explained about 19.5% of the overall heterogeneity, with children showing the strongest association.

### Publication bias assessment

**Figure 2:**
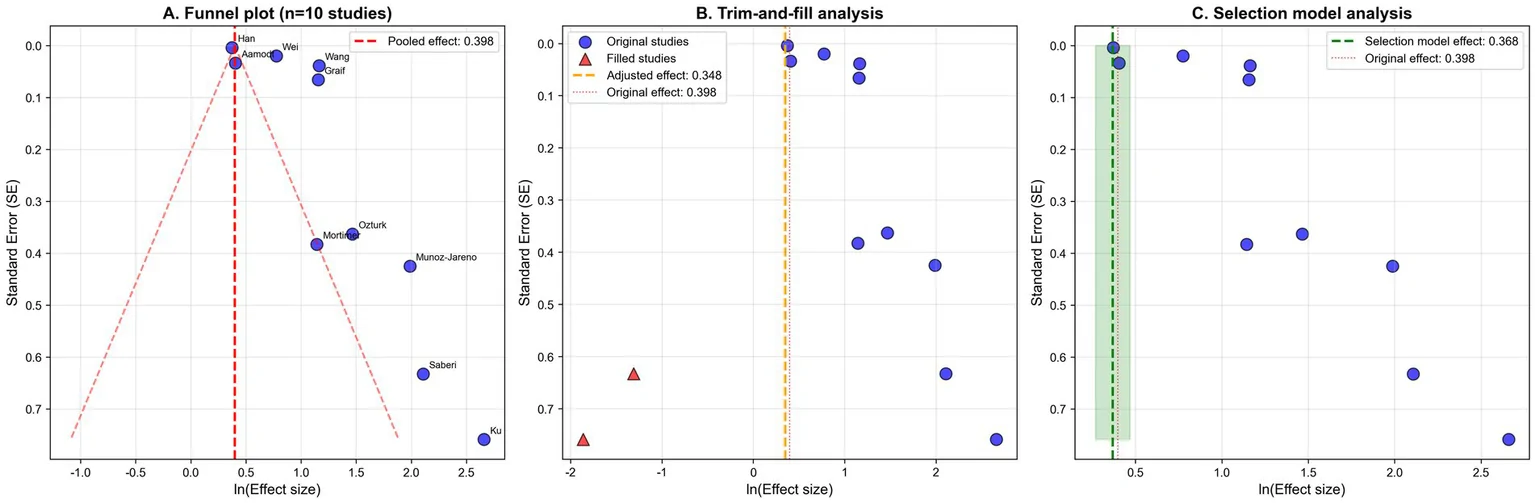
Funnel plot assessing publication bias. **(A)** Funnel plot of included studies. **(B)** Trim-and-fill imputation of potentially missing studies and adjusted pooled effect. **(C)** Selection-model analysis with bias-adjusted pooled effect.

As shown in Figure 2, the funnel plot showed mild asymmetry (Figure 2A), suggesting that selective publication of small-sample studies may have occurred. After imputing two missing studies using the trim-and-fill method (Figure 2B), the combined effect size decreased slightly but remained statistically significant. Selection model analysis (Figure 2C) also showed that the adjusted effect size was slightly smaller than the original result, but in the same direction. These results suggest that publication bias may have slightly overestimated the magnitude of the effect, but do not alter the overall conclusions.

### Sensitivity analyses

Sensitivity analyses revealed that the pooled odds ratio (OR) varied when different criteria were applied (Supplementary Figure S2A). Specifically, the original pooled OR (including all studies) was 2.75 (95% CI: 1.80–4.19). Exclusion of cross-sectional studies yielded a similar OR of 2.60 (95% CI: 1.33–5.07). Analysis restricted to large-sample studies resulted in a slightly lower OR of 1.92 (95% CI: 1.42–2.59), while relying solely on unadjusted ORs produced a higher OR of 5.35 (95% CI: 2.80–10.22).

The leave-one-out analysis indicated that excluding any single study resulted in pooled OR estimates ranging narrowly from 2.42 (95% CI: 1.65–3.56) to 3.05 (95% CI: 1.93–4.82) (Supplementary Figure S2B). This indicates that no single study disproportionately influenced the overall pooled result, supporting the stability of the findings.

### Sources of heterogeneity

In multivariate meta-regression and τ^2^ decomposition analyses (Supplementary Figures S3A–D), study design, age group, and sample size all showed some tendency to explain the pooled effect. Although the overall R^2^ was low (<10%), the results showed that adult studies tended to report higher effect sizes, while cohort studies and studies with large sample sizes had relatively lower effect sizes. τ^2^ decomposition results indicated that study design contributed approximately 18.6% of the heterogeneity, age group approximately 27.9%, and region approximately 7%, leaving approximately one-third of the remaining heterogeneity unexplained. This suggests that the differences between the included studies primarily stem from study design and population characteristics.

A cumulative meta-analysis (Supplementary Figure S2C) showed that early, small-sample studies (1990s–2000s) had larger pooled effects and wider confidence intervals. With the inclusion of larger cohort and case–control studies after 2015, the pooled effect gradually decreased and stabilized, ultimately settling at approximately 2–3 times the risk. This trend suggests that as evidence accumulates, estimates of the association between AR and migraine become more robust, reducing the potential overestimation caused by early, small-sample studies.

### Absolute effects

Based on scenario conversions based on baseline prevalence in different populations (Supplementary Figures S4A–D), the absolute risk difference for migraine associated with AR ranged from approximately 23 additional cases per 1,000 population in low-prevalence populations to 82 additional cases per 1,000 population in high-prevalence populations. The corresponding population attributable fractions (PAFs) ranged from 6.8 to 18.1%. The converted “number needed to treat” (NNT-like) values ranged from 12 to 44. This theoretical estimate suggests that up to one-third of migraine cases might be associated with AR. Similarly, the derived ‘NNT-like’ value should be interpreted strictly as a speculative metric of association strength rather than a predictor of actual treatment benefit. These figures do not imply that eliminating AR will definitively prevent migraine, given the non-randomized nature of the evidence.

## Discussion

### Overview of main findings

This systematic review and meta-analysis included ten observational studies involving more than 4.8 million participants, and we found a significant positive association between allergic rhinitis (AR) and an increased risk of migraine, with an overall pooled odds ratio (OR) of 2.75 (95% CI, 1.80–4.19). Sensitivity analysis showed that this association remained stable across a variety of study designs, age groups, and methodologies, confirming the robustness of our findings.

Our results are consistent with previous epidemiological and clinical studies on the association between allergic diseases and migraine risk. A recent meta-analysis involving more than 14.9 million participants also reported an increased risk of migraine in patients with allergic diseases, especially those with allergic rhinitis, with an OR comparable to our findings (OR = 2.16) (20, 32). In addition, observational studies from different geographic regions, including Europe, Asia, and North America, have consistently shown a higher prevalence of migraine in AR patients than in healthy controls, further strengthening the robustness of our findings (32, 33).

Detailed subgroup analyses further confirmed the robustness of our findings. For example, our study showed that this association persisted across different study designs, including cohort studies, case–control studies, and cross-sectional studies, which is consistent with the results of previous systematic reviews that showed that the association remained stable regardless of the study method (22, 32–35). In addition, our age-stratified analysis showed that the association was stronger in the pediatric population (OR = 3.69, 95% CI: 1.40–9.71) compared with adults (OR = 1.63, 95% CI: 0.96–2.74), suggesting that there may be age-related susceptibility to inflammatory and nervous system interactions between AR and migraine (20, 36).

### Potential biological mechanisms

Several biological mechanisms have been proposed to explain the association between allergic rhinitis and migraine. One of the main mechanisms involves calcitonin gene-related peptide (CGRP)-mediated neurogenic inflammation. CGRP is a potent vasodilator and inflammatory mediator that has been implicated in the pathophysiology of migraine, and elevated CGRP levels have been demonstrated in patients with AR, suggesting that common inflammatory pathways exist between the two diseases (31, 37, 38). AR-specific inflammatory mediators, including histamine and cytokines such as IL-1β, IL-6, and TNF-*α*, can enhance the sensitivity of trigeminal pain receptors and exacerbate migraine attacks by enhancing vasodilation, neurogenic inflammation, and central sensitization (32, 39, 40).

In addition, genetic susceptibility has also been investigated as a possible factor in this association. However, a recent Mendelian randomization analysis suggested that the relationship between AR and migraine may be primarily due to environmental or immune-mediated mechanisms rather than genetic factors (38, 41, 42). This is consistent with our findings, as despite methodological heterogeneity, a significant association was still demonstrated after carefully designed subgroup analyses and adjustment for confounders.

### Clinical and therapeutic implications

In clinical practice, recognizing AR as a risk factor for migraine may have important implications for patient management. Several observational studies and small clinical trials have reported beneficial effects of standard AR treatments (e.g., antihistamines and intranasal corticosteroids) on migraine frequency and severity, which may highlight the potential for therapeutic synergy (42, 43). In addition, emerging biologic therapies targeting CGRP and its receptors have shown efficacy in preventing migraine and may offer dual therapeutic benefits to patients with comorbid AR and migraine by reducing neurogenic inflammation.

The significant difference in risk estimates between pediatric and adult populations may be attributed to several biological and clinical factors. In children, the immune system is still undergoing maturation, which may lead to more pronounced neuroinflammatory responses to allergic triggers (44). Furthermore, the concept of ‘early-onset neural priming’ suggests that allergic exposure during critical developmental windows could sensitize the trigeminal vascular system more intensely in younger individuals. In contrast, adults often present with greater diagnostic overlap due to comorbidities (e.g., tension-type headache, sinusitis), which may dilute the observed association. These factors collectively contribute to the heterogeneity observed in our meta-regression analysis.

### Limitations and strengths

This study has several limitations. First, there was significant heterogeneity among the included studies (I^2^ = 99%), which reflected differences in diagnostic criteria, patient populations, and study designs. Although subgroup and sensitivity analyses mitigated this heterogeneity to some extent, residual variability was still inevitable. Second, all included studies were observational, which inherently limits the ability to draw definitive causal conclusions. Third, some analyses were based on administrative health records or self-reported diagnostic criteria, which may introduce misclassification bias. Finally, While both metrics (OR and HR) suggest a positive association, we acknowledge that they represent different epidemiological estimands and should be interpreted with caution regarding causality. Despite these limitations, our study has significant strengths. Comprehensive inclusion of studies with large sample sizes (e.g., national registries) enhances the generalizability of our findings. Rigorous methodology, including comprehensive sensitivity analyses and detailed subgroup analyses, further enhances the robustness of our conclusions.

To further elucidate the relationship between allergic rhinitis (AR) and migraine, future studies should focus on prospective longitudinal studies and randomized controlled trials to evaluate whether effective AR management can prospectively reduce migraine incidence. Mechanistic studies exploring the precise molecular interactions between neurogenic inflammation and allergic immune responses will further elucidate the pathogenic pathways. Finally, genetic and epigenetic studies using large-scale genomic databases may further elucidate potential shared biological vulnerabilities.

## Conclusion

In summary, this systematic review and meta-analysis suggest a consistent association between allergic rhinitis and an increased odds/risk of migraine. However, given the extreme heterogeneity and the observational nature of the data, these findings should be interpreted with caution and do not establish causality. While our results highlight a potential link that clinicians may consider, further high-quality prospective studies are needed before routine screening recommendations can be firmly established.

## Data Availability

The original contributions presented in the study are included in the article/Supplementary material, further inquiries can be directed to the corresponding author.

## Supplementary material

SUPPLEMENTARY FIGURE S1 **(A)** Forest plot of the association using raw Odds Ratios from all applicable studies. **(B)** Forest plot of the association using Hazard Ratios from cohort studies only. **(C)** Forest plot of the association after converting Odds Ratios to Relative Risks to correct for overestimation bias.

SUPPLEMENTARY FIGURE S2 **(A)** Comprehensive sensitivity analysis (by different exclusion criteria). **(B)** Leave-one-out sensitivity analysis. **(C)** Cumulative meta-analysis (chronological order by publication year).

SUPPLEMENTARY FIGURE S3 Meta-regression and τ^2^ decomposition **(A)** Univariable meta-regression by study design. **(B)** Univariable meta-regression by age group. **(C)** Univariable meta-regression by sample size (log-scale). **(D)** τ^2^ variance decomposition and partial R^2^ for key moderators.

SUPPLEMENTARY FIGURE S4 Absolute effect translations of the pooled relative risk **(A)** Risk difference (per 1,000) under low baseline prevalence scenario. **(B)** Risk difference under high baseline prevalence scenario. **(C)** Population attributable fraction (PAF) across prevalence scenarios. **(D)** NNT-like values across prevalence scenarios.
