# Supplementary figures and images for: A systematic review and meta-analysis on the risk of migraine in patients with allergic rhinitis
Source: Front Med (Lausanne). 2026 Apr 2;13:1766176. doi: 10.3389/fmed.2026.1766176 (PMC13082992; doi:10.3389/fmed.2026.1766176)

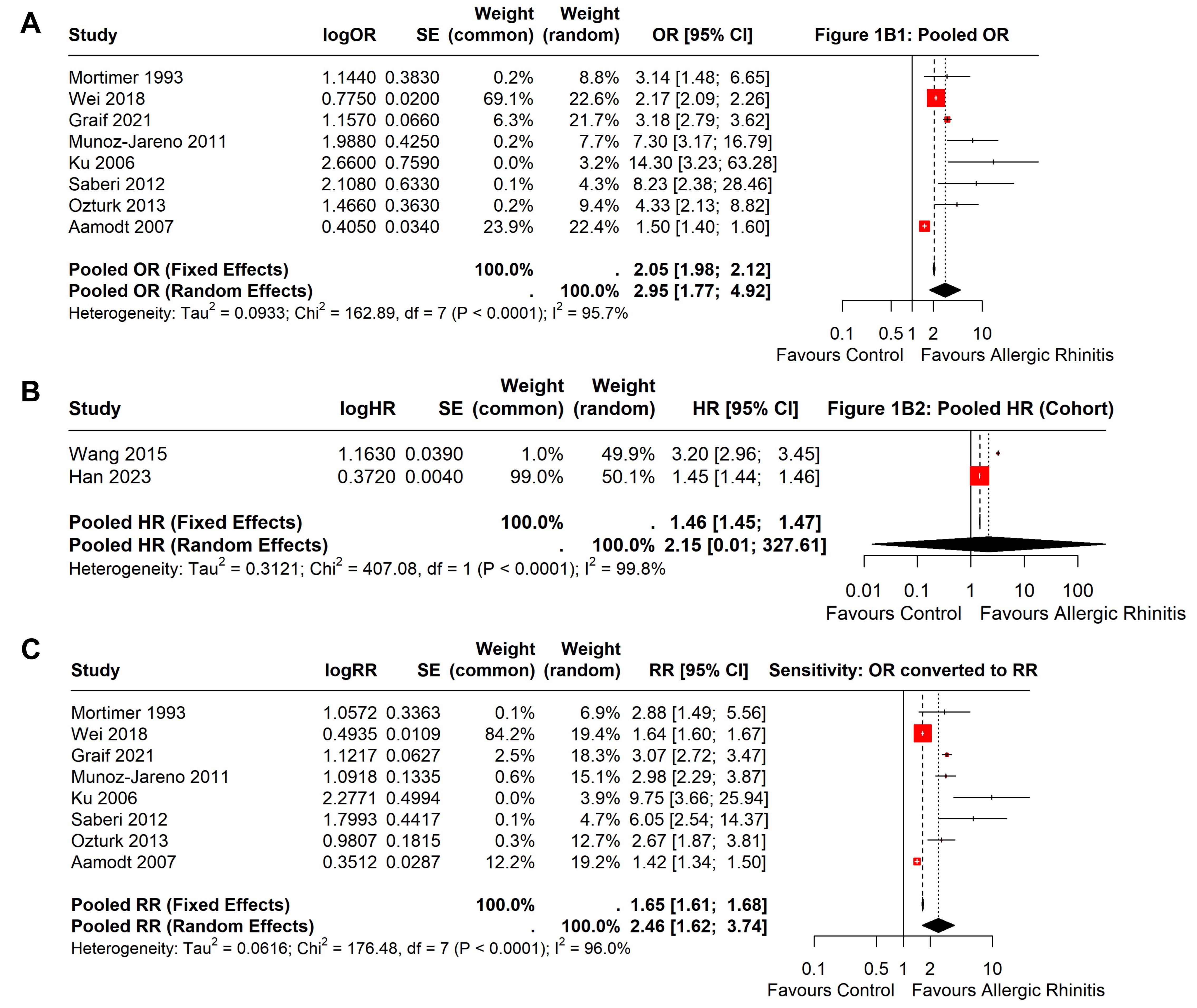

Supplement: SUPPLEMENTARY FIGURE S1 — (A) Forest plot of the association using raw Odds Ratios from all applicable studies. (B) Forest plot of the association using Hazard Ratios from cohort studies only. (C) Forest plot of the association after converting Odds Ratios to Relative Risks to correct for overestimation bias. [file image_1.jpeg]

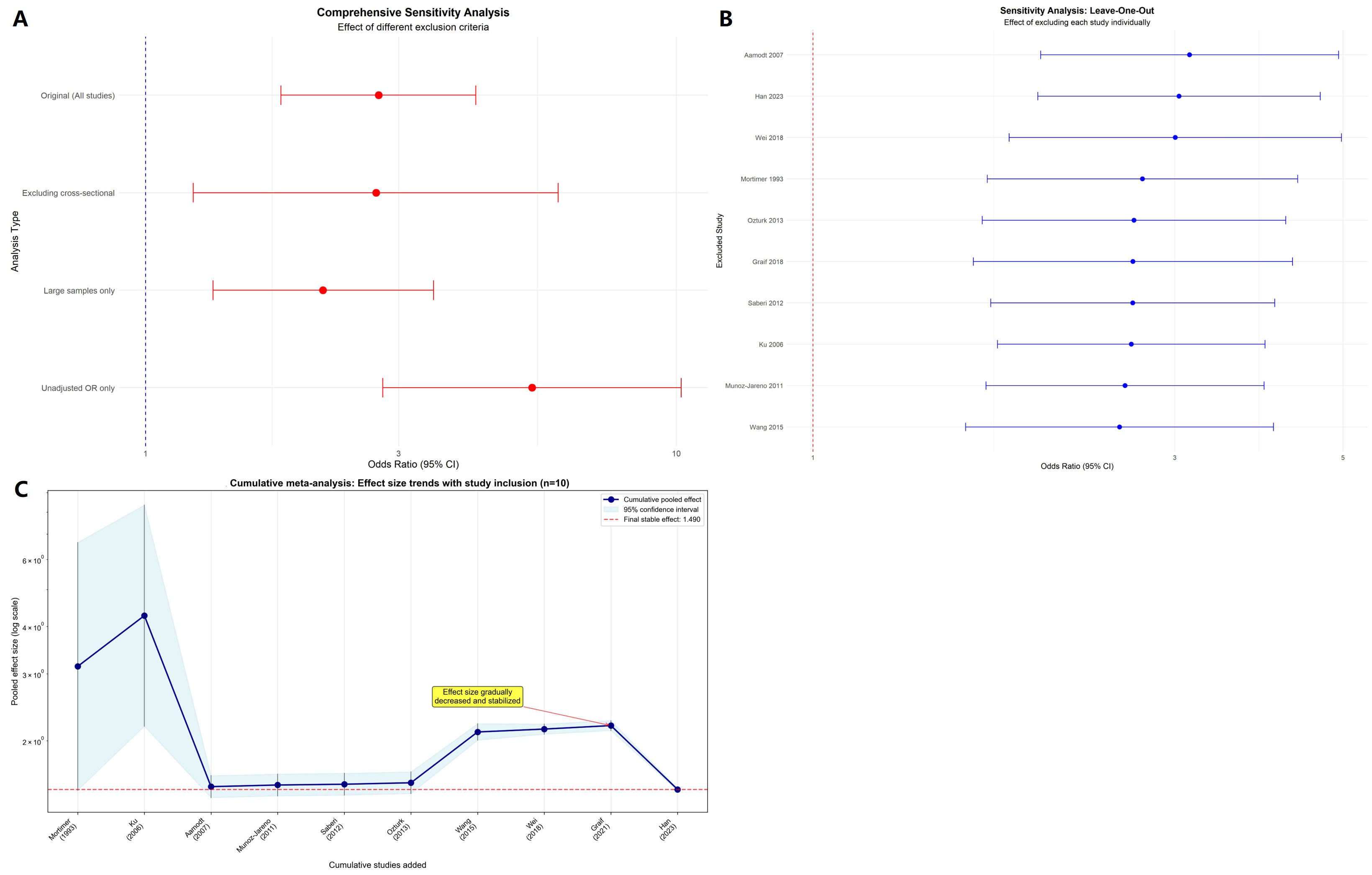

Supplement: SUPPLEMENTARY FIGURE S2 — (A) Comprehensive sensitivity analysis (by different exclusion criteria). (B) Leave-one-out sensitivity analysis. (C) Cumulative meta-analysis (chronological order by publication year). [file image_2.jpeg]

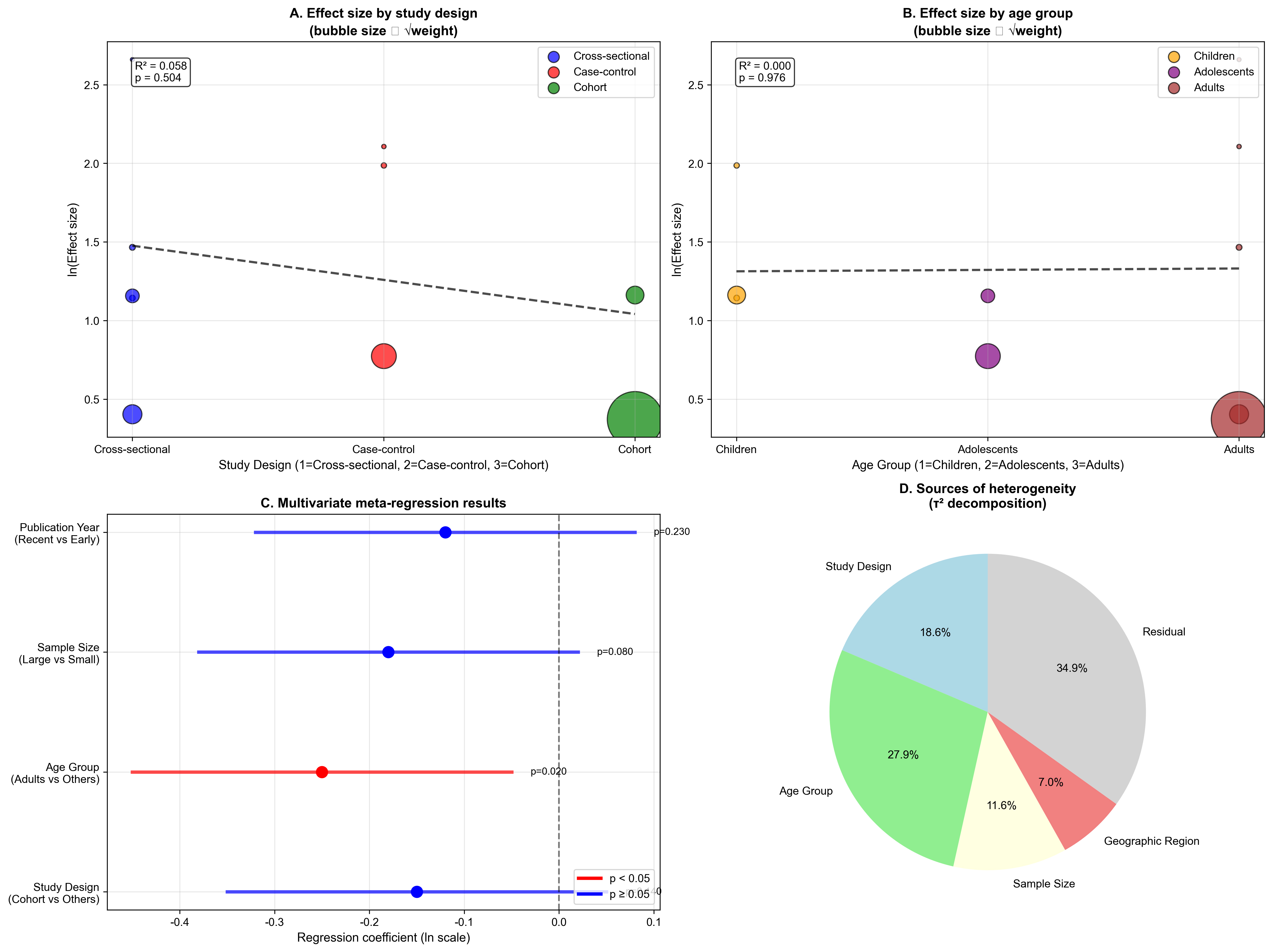

Supplement: SUPPLEMENTARY FIGURE S3 — Meta-regression and τ2 decomposition (A) Univariable meta-regression by study design. (B) Univariable meta-regression by age group. (C) Univariable meta-regression by sample size (log-scale). (D) τ2 variance decomposition and partial R2 for key moderators. [file image_3.jpeg]

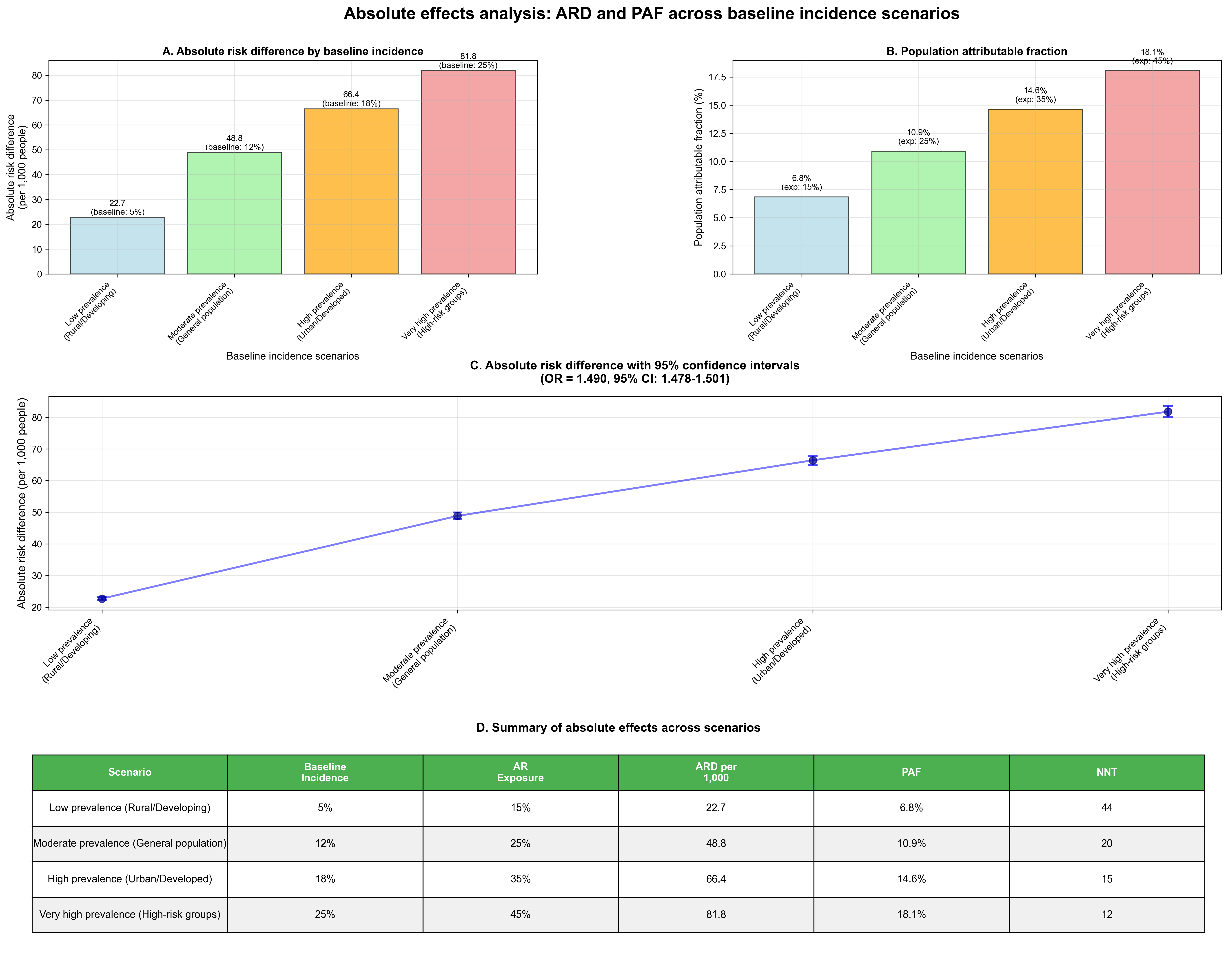

Supplement: SUPPLEMENTARY FIGURE S4 — Absolute effect translations of the pooled relative risk (A) Risk difference (per 1,000) under low baseline prevalence scenario. (B) Risk difference under high baseline prevalence scenario. (C) Population attributable fraction (PAF) across prevalence scenarios. (D) NNT-like values across prevalence scenarios. [file image_4.jpeg]
